# Development of *Leishmania* (*Mundinia*) in guinea pigs

**DOI:** 10.1186/s13071-020-04039-9

**Published:** 2020-04-08

**Authors:** Tomas Becvar, Padet Siriyasatien, Paul Bates, Petr Volf, Jovana Sádlová

**Affiliations:** 1 Department of Parasitology, Faculty of Science, Charles University, Prague, Czech Republic; 2 Vector Biology and Vector Borne Disease Research Unit, Department of Parasitology, Faculty of Medicine, Chulalongkorn University, Bangkok, Thailand; 3 Division of Biomedical and Life Sciences, Faculty of Health and Medicine, Lancaster University, Lancaster, UK

**Keywords:** *Leishmania*, *Mundinia*, Guinea pig, *Leishmania enriettii*, *Leishmania martiniquensis*, *Leishmania orientalis*, *Leishmania macropodum*, Animal model

## Abstract

**Background:**

Leishmaniasis is a human and animal disease caused by parasites of the genus *Leishmania*, which is now divided into four subgenera, *Leishmania*, *Viannia*, *Sauroleishmania* and *Mundinia*. Subgenus *Mundinia*, established in 2016, is geographically widely dispersed, its distribution covers all continents, except Antarctica. It consists of 5 species; *L. enriettii* and *L. macropodum* are parasites of wild mammals while *L. martiniquensis*, *L. orientalis* and an unnamed *Leishmania* sp. from Ghana are infectious to humans. There is very little information on natural reservoir hosts and vectors for any *Mundinia* species.

**Methods:**

Experimental infections of guinea pigs with all five *Mundinia* species were performed. Animals were injected intradermally with 10^7^ culture-derived promastigotes into both ear pinnae. The courses of infections were monitored weekly; xenodiagnoses were performed at weeks 4 and 8 post-infection using *Lutzomyia migonei*. The distribution of parasites in different tissues was determined *post-mortem* by conventional PCR.

**Results:**

No significant differences in weight were observed between infected animals and the control group. Animals infected with *L. enriettii* developed temporary lesions at the site of inoculation and were infectious to *Lu. migonei* in xenodiagnoses. Animals infected with *L. martiniquensis* and *L. orientalis* developed temporary erythema and dry lesions at the site of inoculation, respectively, but were not infectious to sand flies. Guinea pigs infected by *L. macropodum* and *Leishmania* sp. from Ghana showed no signs of infection during experiments, were not infectious to sand flies and leishmanial DNA was not detected in their tissue samples at the end of experiments at week 12 post-inoculation.

**Conclusions:**

According to our results, guinea pigs are not an appropriate model organism for studying *Mundinia* species other than *L. enriettii.* We suggest that for better understanding of *L.* (*Mundinia*) biology it is necessary to focus on other model organisms.
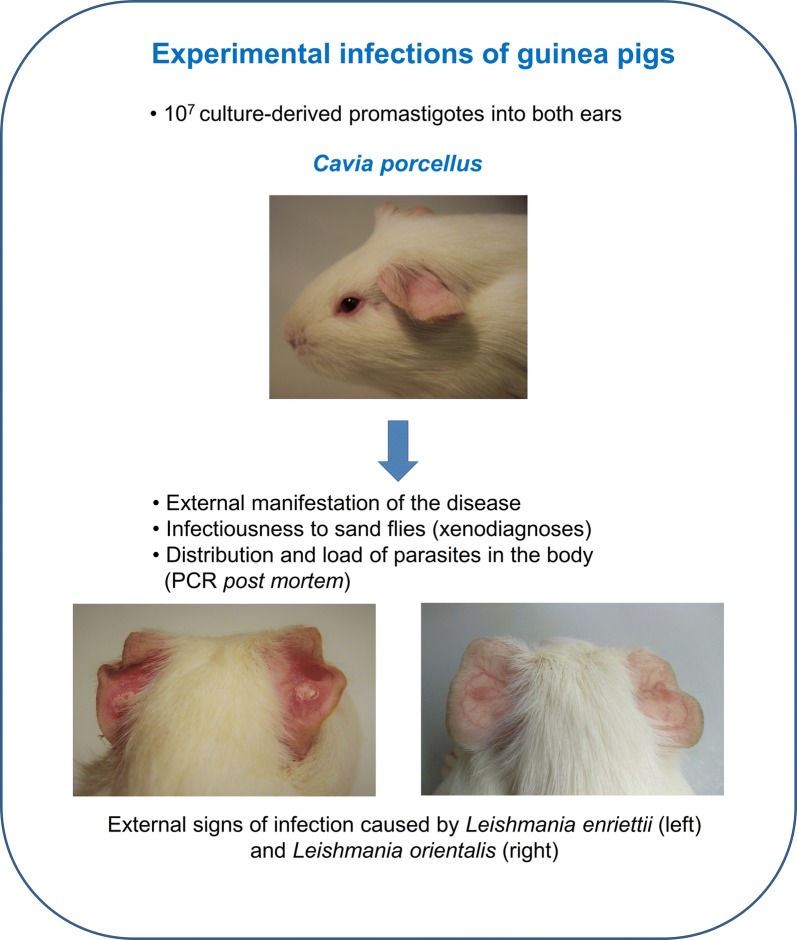

## Background

Leishmaniases are vector-borne diseases whose etiological agents are protozoan parasites of the genus *Leishmania* (Kinetoplastida: Trypanosomatidae). Known previously as the *L. enriettii* complex, the subgenus *Mundinia* was established recently and currently contains 5 species: *L. enriettii*, *L. macropodum*, *L. orientalis*, *L. martiniquensis* and an unnamed *Leishmania* sp. from Ghana [1–3]. According to phylogenetic analyses, this subgenus is the first to branch from the other *Leishmania* subgenera, indicating that species of this subgenus are likely to represent the most ancient and divergent group of species within the *Leishmania* [2, 4]. The geographical distribution of *Mundinia* species covers all continents except Antarctica, which can be explained by the formation of individual species from their common ancestor after the breakup of Gondwana [2].

Many important details of the biology of these parasites are unknown. The identity of the insect vectors responsible for transmission of *L.* (*Mundinia*) has not been confirmed for any species yet. It has been assumed that these parasites, similar to other *Leishmania*, would be transmitted by sand flies of the genus *Phlebotomus* and/or *Sergentomyia* in the Old World and *Lutzomyia* in the New World (Diptera: Phlebotominae), and this may be the case. Recently, however, *Forcipomyia* (*Lasiohelea*) biting midges (Diptera: Ceratopogonidae) were reported as likely vectors of *L. macropodum* in Australia [5], and laboratory experiments have revealed a high susceptibility of *Culicoides sonorensis* to *L. enriettii* [6]. The observations raise the possibility of non-sand fly vectors for at least some of the *Mundinia*.

Similarly, there is little current information on the natural mammalian reservoir hosts for these parasites. *Leishmania enriettii*, is a parasite that has only ever been found in domestic guinea pigs (*Cavia porcellus*) in Brazil, first isolated in the 1940s [7]. The natural host of *L. enriettii* is not known although often assumed to be a wild rodent of some kind. *Leishmania macropodum* is a parasite first isolated from red kangaroos in Australia, but from a game park in a region where these animals are not found [5]. There is evidence of *L. macropodum* infection in three other species of Australian macropods, which are more likely to be the true host(s) of this parasite [7]. On the other hand, human cases have been described with *L. martiniquensis*, *L. orientalis* and *Leishmania* sp. from Ghana. *Leishmania martiniquensis* was first isolated from a HIV positive man on Martinique Island in 1992 [8]. According to recent findings, this *Leishmania* species has a worldwide distribution with single or multiple cases reported from various continents where these parasites were isolated from various hosts such as horses, cows and humans [9–12]. *Leishmania orientalis* was formally described in 2018 [13]; in the past it was reported as “*L. siamensis*” [9, 14] but this name is a *nomen nudum* and should not be used anymore. *Leishmania* sp. from Ghana is a species causing cutaneous leishmaniasis in the Volta region in Ghana [4]. The last two species were not isolated from any mammalian species, except humans, and the identity of their reservoir hosts remains enigmatic.

Since very little is known about biology of these neglected parasites, the aim of our study was the establishment of model host organisms, which would enables testing their behaviour and properties in a mammalian host. Here we present results of experimental infections in guinea pigs with all five known *L.* (*Mundinia*) species.

## Methods

### Parasites and guinea pigs

*Leishmania enriettii* (MCAV/BR/45/LV90), *L. macropodum* (MMAC/AU/2004/AM-2004), *Leishmania* sp. from Ghana (MHOM/GH/2012/GH5), *L. orientalis* (MHOM/TH/2014/LSCM4) and two strains of *L. martiniquensis* (MHOM/MQ/1992/MAR1 and MHOM/TH/2011/CU1) were used. Parasites were maintained at 23 °C in M199 medium supplemented with 10% fetal calf serum (Gibco, Prague, Czech Republic), 1% BME vitamins (Sigma-Aldrich, Prague, Czech Republic), 2% sterile urine and 250 μg/ml amikacin (Amikin, Bristol-Myers Squibb, Prague, Czech Republic). In our laboratory both strains were maintained in a cryobank with 2–3 sub-passages *in vitro* before experimental infections of guinea pigs and no passages in animals were performed. Before experimental infection, parasites were washed by centrifugation (6000×*g* for 5 min) and resuspended in saline solution.

Female guinea pigs (Dunkin-Hartley) originating from AnLab (Prague, Czech Republic) were maintained in groups of 2 specimens in T4 boxes (58 × 37 × 20 cm); Velaz (Prague, Czech Republic) equipped with bedding (German Horse Span; Pferde, Prague, Czech Republic), breeding material (Woodwool) and hay (Krmne smesi Kvidera, Spalene Porici, Czech Republic), provided with a feed mixture V2233 Ms-H Guinea Pig maintenance (AnLab) and water *ad libitum*, with a 12 h light/12 h dark photoperiod, temperature of 22–25 °C and relative humidity of 40–60%. At the beginning of experiments the average weight of animals was 499 g and average age was 7 weeks.

### Infection and xenodiagnoses of guinea pigs

Eighteen guinea pigs (*Cavia porcellus*) anaesthetized with ketamin/xylazin (37.5 mg/kg and 1.5 mg/kg, respectively) were injected with 10^7^ stationary-stage promastigotes in 5 µl of sterile saline intradermally into the ear pinnae of both ears. The course of infection was recorded weekly. Three animals inoculated with the same volume of saline solution were used as a control for external signs of infection.

Xenodiagnoses were performed at weeks 4 and 8 post-infection (pi) using the permissive vector *Lutzomyia migonei* [15]. Five to six-day-old *Lu*. *migonei* were placed into plastic vials covered by fine nylon mesh and allowed to feed on the ear pinnae of anaesthetized animals. Engorged individuals were maintained for two days at 25 °C and then stored in tissue lysis buffer (Roche, Prague, Czech Republic) at -20 °C in pools of 5 females for subsequent PCR. Altogether 192 sand fly pools were tested.

At the end of the experiments, 12 weeks post-infection (pi), the hosts were euthanized, dissected and tissues from ears, paws, ear-draining lymph nodes, spleens, livers and blood were stored at − 20 °C for subsequent PCR.

### Conventional PCR

DNA extraction from vectors and animal tissues was performed using the High Pure PCR Template Preparation Kit (Roche) according to the manufacturer’s instructions. The total DNA was used as a template for PCR amplification with the primers for a 246 bp long ITS1 sequence (forward primer 5′-AGA TTA TGG AGC TGT GCG ACA A-3′ and reverse primer 5′-TAG TTC GTC TTG GTG CGG TC-3′). Reactions were performed using EmeraldAmp^®^ GT PCR Master Mix and cycling conditions were as follows: step 1, 94 °C for 3 min 30 s; step 2, 94 °C for 30 s; step 3, 60 °C for 30 s; step 4, 72 °C for 20 s; step 5, 72 °C for 7 min; followed by cooling at 12 °C. Steps 2–4 were repeated 35 times. Samples were analysed using 1% agarose gels.

### Statistical analysis

Statistical analyses were carried out using R software ((http://cran.r-project.org/). Correlation of animal weight in different groups (infected and non-infected) and time was tested by co-variance analysis.

## Results

No significant differences in weight were observed between infected animals and the control group (*L. macropodum*, *P* = 0.70; *L. enriettii*, *P* = 0.12; *L. martiniquensis* MAR1, *P* = 0.77; *L. martiniquensis* CU1, *P* = 0.12; *Leishmania* sp. from Ghana, *P* = 0.20; *L. orientalis*, *P* = 0.11; see Additional file 1: Table S1).

**Fig. 1:**
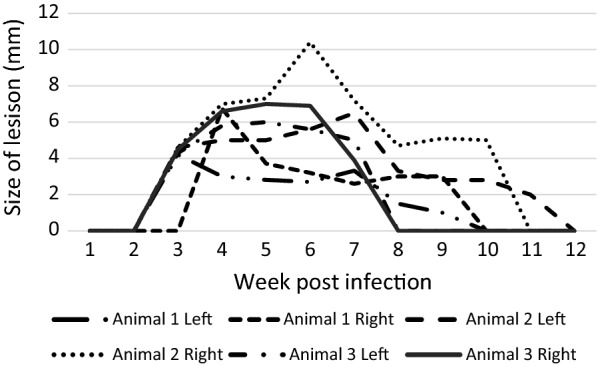
Development of lesions in guinea pigs infected with *L. enriettii*

**Fig. 2:**
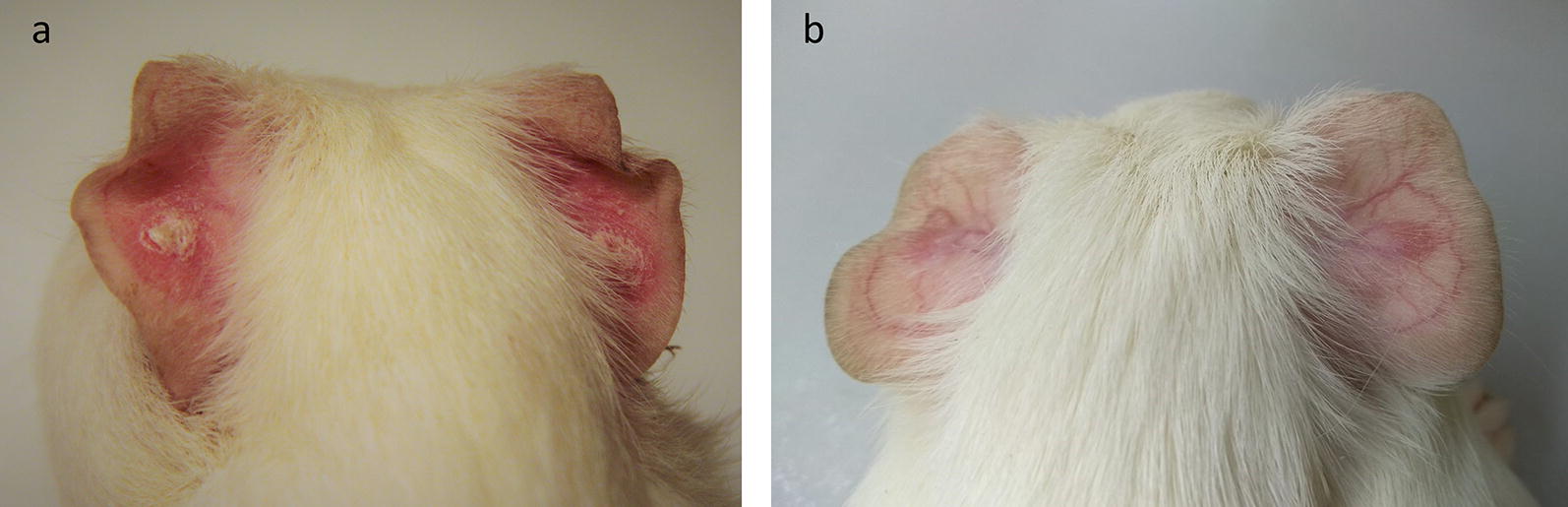
Dry lesions observed in animals infected with *L. enriettii* (**a**) and nodules transforming to dry lesions in animals infected by *L. orientalis* (**b**); both at week 5 pi

Development of dry lesions was observed in animals infected with *L. enriettii*. The lesions appeared on ear pinnae (the site of inoculation) by week 2–3 pi, increased in size through to week 5–6 pi and then healed, completely disappearing between weeks 8–12 pi (Figs. 1, 2a). Animals were efficiently infectious to sand flies on week 4 pi (9/16 positive pools) while infectiousness was reduced by week 8 pi (1/16 positive pools).

In animals infected with *L. martiniquensis* (MAR1), temporary erythema was observed at the site of inoculation between weeks 4–8 pi, but no lesions developed and animals were not infectious to sand flies (0/32 positive pools).

Erythema at the site of inoculation was also observed in guinea pigs infected with *L. orientalis.* The erythematic spot appeared by week 3–4 pi. The spot nodulated, and the nodules subsequently transformed into dry lesions surrounded by a purple skin macula (Fig. 2b, Additional file 2: Table S2). The lesions healed by week 7–8 pi. However, animals were not infectious to sand flies (0/32 positive pools) and no leishmanial DNA was detected in any of the tested tissue samples.

Guinea pigs infected with *L. macropodum*, *Leishmania* sp. from Ghana and *L. martiniquensis* (CU1) did not show any external signs of infection during the whole experiment, animals were not infectious to sand flies (0/96 positive pools consisting of 5 blood-fed females each), and leishmanial DNA was not detected in any of tissue samples collected by the end of experiment on week 12 pi.

## Discussion

Leishmaniases of men and animals caused by *L.* (*Mundinia*) species are emerging all over the world. The diseases in humans are characterized by symptoms varying from self-healing skin lesions [2, 12] to visceral forms. The latter prevail in HIV-positive patients [8, 16] but was also observed in immunocompetent humans [12, 17]. Very little is known about the life-cycle of these ancient and neglected species and an appropriate animal model is necessary for closer understanding of their biology.

Guinea pigs were chosen for the experimental model in our study as they are the only known non-human mammalian hosts of *L.* (*Mundinia*) species, except for kangaroos, cows and horses [6, 7, 9, 10, 18], which are not practicable for most laboratory investigations. *Leishmania enriettii* was repeatedly isolated from domestic guinea pigs from various localities in Brazil [6, 19]. Interestingly, individual cases were separated by long time periods, which does not agree with the fact that guinea pigs are popular pets, and according to several studies, they are very susceptible to infection [5, 20, 21]. We suggest that this rare incidence may have two different explanations. First, the prevalence of infection is actually much higher, but the owners of infected guinea pigs do not take them for veterinary checks, therefore, parasites are not isolated. Alternatively, guinea pigs are only incidental hosts and the primary reservoir hosts (and primary insect vectors) are not present in close vicinity to households. In this case, secondary vectors and/or reservoirs may be temporarily involved in transmission to domestic localities and domestic guinea pigs.

Our experiments confirmed the susceptibility of guinea pigs to *L. enriettii*. All infected animals showed development of typical ear lesions and the animals were infectious to sand flies. The numbers of positive sand flies were significantly higher at week 4 pi than at the later time interval, week 8 pi. This decrease of infectivity was also observed previously by Seblova et al. [6]. At week 12 pi, the animals did not show any more external signs of infection and *Leishmania* DNA was not detected in any of the examined tissue samples. Spontaneous healing of lesions was observed also by Paranaiba et al. [22]. In their experiments initiated by intradermal inoculation of 10^5^ promastigotes, a different virulence between the two strains used was observed. The Cobaia strain did not develop any lesions, while strain L88 developed lesions that were growing by weeks 4–6 pi, and then diminution of lesions was observed until the end of the experiment. The authors also described development of larger lesions in groups where sand fly salivary glands were added to the inoculum.

However, the virulence of the *L. enriettii* parasite strain and the presence of sand fly salivary glands are not the sole factors influencing the degree of pathogenicity for guinea pigs. The outcome of infections is also dependent on the method of their initiation, i.e. on parasite numbers and stages (amastigotes *vs* promastigotes) used as an inoculum. Thomaz-Soccol et al. [23] described the development of serious symptoms of disease, such as the dissemination of parasites and subsequent death of all tested animals, when the inoculum consisted of amastigotes of a strain identical to L88 (according to isoenzyme analyses). Wide dissemination of *L. enriettii* in animals was also observed by Paraense et al. [20], who infected guinea pigs with amastigotes from lesion homogenates, and by Seblova et al. [6] who infected animals with 10^7^ culture derived promastigotes.

Development of *L. enriettii* has also been tested in hamsters (*Mesocricetus auratus*), where infections were characterized by the development of temporary lesions at the site of inoculation and their subsequent healing [5, 24]. In experiments with wild guinea pigs (*Cavia aperea*), rhesus macaques and dogs [7], no animals showed any signs of infection, so domestic guinea pigs remain the best laboratory model for *L. enriettii* at present.

Here, we compared the susceptibility of guinea pigs to four other *L.* (*Mundinia*) species. The infections were lost in animals infected with *L. macropodum*, *Leishmania* sp. from Ghana and *L. martiniquensis* strain CU1. Animals infected with a second *L. martiniquensis* strain, MAR1, and with *L. orientalis* developed only temporary changes on the ears and the animals were not infectious to sand flies. However, PCR analysis showed no presence of leishmanial DNA by week 12 pi in any of the tested samples. We suggest that *L. martiniquensis* and *L. orientalis* are capable of temporary survival at the site of inoculation, but they cannot disseminate to other tissues of guinea pigs.

We suggest that for a better understanding of *L.* (*Mundinia*) biology it is necessary to focus on other model host organisms. The first choice could be BALB/c mice or hamsters as the most common animal models for research used with many *L*. (*Leishmania*) and *L*. (*Viannia*) species. Infections of these standard laboratory animals with their controlled genetic background may bring valuable information. Alternatively, genetically polymorphic models like wild rodents mimicking natural hosts could be used. These less common models can allow a better understanding of the dynamics of infection and host-parasite relationships related more closely to the situation in the wild [24]. On the other hand, when infected with *L. enriettii* and *L. orientalis*, guinea pigs could serve as a potential model for spontaneous healing, which could be informative for the design of vaccines.

## Conclusions

Experimental infections showed that guinea pigs are not a good animal model for the subgenus *Mundinia*, with exception of *L. enriettii*. All other *Mundinia* species studied, *L. orientalis*, *L. martiniquensis*, *L. macropodum* and *L*. (*Mundinia*) sp. from Ghana, were not able to develop infections transmissible to sand flies.

## Supplementary information

**Additional file 1: Table S1.** Weight gain of guinea pigs during the experiment.

**Additional file 2: Table S2.** External signs of infection on ears of infected guinea pigs during the experiment. *Abbreviations*: E, erythema; N, nodulus; DL, dry lesion.

## Data Availability

All the data are included within the article and its additional files.

## Abbreviations

PCR: polymerase chain reaction
pi: post-infection
