# Supplementary material for: Development of Leishmania (Mundinia) in guinea pigs
Source: Parasit Vectors. 2020 Apr 8;13:181. doi: 10.1186/s13071-020-04039-9 (PMC7140393; doi:10.1186/s13071-020-04039-9)
Supplement: Supplementary file 1 — Additional file 1: Table S1. Weight gain of guinea pigs during the experiment. [file 13071_2020_4039_MOESM1_ESM.docx]

**Additional file 1: Table S1.** Weight gain of guinea pigs during the experiment.

| *Leishmania* species | No. of animal | Weeks post infection | | | | | | | | | | | |
| --- | --- | --- | --- | --- | --- | --- | --- | --- | --- | --- | --- | --- | --- |
|  |  | 1 | 2 | 3 | 4 | 5 | 6 | 7 | 8 | 9 | 10 | 11 | 12 |
| *L*. *martiniquensis* Mar1 | 1 | 464 | 497 | 548 | 571 | 625 | 693 | 713 | 787 | 780 | 880 | 888 | 919 |
|  | 2 | 479 | 503 | 545 | 586 | 595 | 651 | 653 | 754 | 807 | 829 | 856 | 876 |
|  | 3 | 480 | 512 | 560 | 594 | 614 | 639 | 661 | 755 | 779 | 811 | 823 | 863 |
| *L*. sp. from Ghana | 4 | 467 | 482 | 505 | 545 | 593 | 633 | 667 | 749 | 743 | 802 | 820 | 833 |
|  | 5 | 483 | 505 | 585 | 614 | 634 | 685 | 697 | 751 | 778 | 789 | 753 | 727 |
|  | 6 | 459 | 462 | 500 | 510 | 540 | 598 | 624 | 696 | 734 | 744 | 774 | 797 |
| *L.* *martiniquensis* Cu1 | 7 | 463 | 490 | 542 | 621 | 628 | 651 | 691 | 711 | 710 | 741 | 776 | 792 |
|  | 8 | 442 | 484 | 549 | 586 | 630 | 647 | 692 | 710 | 700 | 730 | 756 | 768 |
|  | 9 | 443 | 562 | 569 | 595 | 656 | 661 | 697 | 710 | 802 | 728 | 765 | 772 |
| *L. orientalis* | 10 | 444 | 535 | 538 | 592 | 630 | 667 | 690 | 763 | 775 | 793 | 808 | 826 |
|  | 11 | 508 | 540 | 590 | 624 | 674 | 704 | 738 | 780 | 805 | 832 | 851 | 853 |
|  | 12 | 463 | 553 | 557 | 600 | 621 | 646 | 670 | 683 | 684 | 710 | 735 | 750 |
| *L. macropodum* | 13 | 569 | 500 | 526 | 557 | 560 | 573 | 616 | 615 | 582 | 633 | 655 | 680 |
|  | 14 | 634 | 567 | 575 | 669 | 721 | 729 | 768 | 792 | 852 | 892 | 920 | 938 |
|  | 15 | 610 | 558 | 558 | 637 | 664 | 667 | 719 | 763 | 799 | 840 | 853 | 887 |
| *L. enriettii* | 16 | 492 | 434 | 492 | 540 | 560 | 574 | 631 | 670 | 700 | 730 | 746 | 763 |
|  | 17 | 540 | 490 | 502 | 470 | 558 | 533 | 562 | 590 | 571 | 647 | 699 | 731 |
|  | 18 | 548 | 438 | 470 | 528 | 551 | 560 | 655 | 653 | 614 | 722 | 766 | 805 |
| Control | 19 | 566 | 526 | 524 | 576 | 571 | 605 | 633 | 659 | 637 | 705 | 736 | 760 |
|  | 20 | 461 | 477 | 513 | 556 | 602 | 647 | 676 | 753 | 769 | 795 | 795 | 836 |
|  | 21 | 459 | 526 | 569 | 591 | 628 | 662 | 692 | 780 | 808 | 854 | 874 | 887 |
