# Supplementary material for: Development of Leishmania (Mundinia) in guinea pigs
Source: Parasit Vectors. 2020 Apr 8;13:181. doi: 10.1186/s13071-020-04039-9 (PMC7140393; doi:10.1186/s13071-020-04039-9)
Supplement: Supplementary file 2 — Additional file 2: Table S2. External signs of infection on ears of infected guinea pigs during the experiment. Abbreviations: E, erythema; N, nodulus; DL, dry lesion. [file 13071_2020_4039_MOESM2_ESM.docx]

**Additional file 2: Table S2.** External signs of infection on ears of infected guinea pigs during the experiment. E, erythema; N, nodulus; DL, dry lesion.

| Week PI | Animals infected with *L. orientalis* | | | | | | | Animals infected with *L. enriettii* | | | | | | | |
| --- | --- | --- | --- | --- | --- | --- | --- | --- | --- | --- | --- | --- | --- | --- | --- |
|  | No. 1 | | No. 2 | | | No. 3 | | No. 1 | | | No. 2 | | | No. 3 | |
|  | Left ear | Right ear | | Left ear | Right ear | Left ear | Right ear | Left ear | Right ear | Left ear | | Right ear | Left ear | | Right ear |
| 1 | - | - | | - | - | - | - | - | - | - | | - | - | | - |
| 2 | - | - | | - | - | - | - | - | - | - | | - | - | | - |
| 3 | - | - | | - | - | E | E | DL  4.0 mm | - | DL  4.6 mm | | DL  4.6 mm | DL  4.3 mm | | DL  4.5 mm |
| 4 | E | E | | N  1.0 mm | N  1.0 mm | N  1.0 mm | N  1.0 mm | DL  3.0 mm | DL  6.7 mm | DL  5.0 mm | | DL  7.0 mm | DL  5.8 mm | | DL  6.6 mm |
| 5 | N  1.0 mm | N  2.5 mm | | - | DL  2.5 mm | DL  4.0 mm | DL  4.0 mm | DL  2.8 mm | DL  3.7 mm | DL  5.0 mm | | DL  7.3 mm | DL  6.0 mm | | DL  7.0 mm |
| 6 | DL  1.0 mm | N  2.0 mm | | - | DL  2.5 mm | DL  3.0 mm | DL  3.0 mm | DL  2.7 mm | DL  3.2 mm | DL  5.6 mm | | DL  10.4 mm | DL  5.6 mm | | DL  6.9 mm |
| 7 | - | - | | - | - | DL  1.0 mm | DL  1.0 mm | DL  3.3 mm | DL  2.6 mm | DL  6.5 mm | | DL  7.2 mm | DL  5.0 mm | | DL  3.9 |
| 8 | - | - | | - | - | - | - | DL  1.0 mm | DL  3.0 mm | DL  3.3 mm | | DL  4.7 mm | - | | - |
| 9 | - | - | | - | - | - | - | - | DL  3.0 mm | DL  2.8 mm | | DL  5.1 mm | - | | - |
| 10 | - | - | | - | - | - | - | - | - | DL  2.8 mm | | DL  5.0 mm | - | | - |
| 11 | - | - | | - | - | - | - | - | - | DL  2.0 mm | | - | - | | - |
| 12 | - | - | | - | - | - | - | - | - | - | | - | - | | - |
